# Parental abuse and subjective wellbeing among Egyptian early emerging adults: a gender-moderated analysis

**DOI:** 10.3389/fpsyg.2026.1849856

**Published:** 2026-09-24

**Authors:** Mona Mohammed Fouad Mohammed El-Sawaf, Nayera Mohamed Shousha

**Affiliations:** 1 Department of Psychological Sciences, College of Education, Qatar University, Doha, Qatar; 2 School of Social Sciences and Humanities, Doha Institute for Graduate Studies, Doha, Qatar; 3 Psychology Department, Cairo University, Giza, Egypt

**Keywords:** abusive parenting, early emerging adults, Egypt, gender differences, subjective wellbeing

## Abstract

**Background:**

Prolonged co-residence with parents, common in Egyptian early emerging adults (18–21 years), can expose them to continued parental abuse—a phenomenon largely overlooked in current non-western literature. Unlike prior studies focusing on retrospective childhood abuse, this research examines ongoing abuse experiences and their relationship with wellbeing, with particular attention to gender differences.

**Methods:**

Using a cross-sectional design, 360 early emerging adults (aged 18–21; 121 men, 239 women) completed validated measures of perceived parental abusive behaviors and subjective wellbeing. Moderation analyses were conducted using Hayes' (2022) PROCESS macro (Model 1), controlling for age, major specialization, and living arrangement.

**Results:**

63.6% of participants reported moderate-to-high levels of parental abuse, with higher prevalence among early emerging adult women (73.7%) compared to early emerging adult men (43.8%), a significant difference. Gender moderated the association between perceived parental abuse and each dimension of subjective wellbeing: life satisfaction (*B* = 0.12, 95% CI [0.07, 0.16], *p* < 0.001), positive affect (*B* = 0.05, 95% CI [0.00, 0.10], *p* = 0.033), and negative affect (*B* = 0.07, 95% CI [0.02, 0.11], *p* = 0.006). Specifically, the association between abuse and life satisfaction was negative among women but positive among men; the negative association between abuse and positive affect was significant among women only; and the positive association between abuse and negative affect was significant in both genders but stronger among men.

**Conclusion:**

These findings highlight the enduring influence of familial dynamics and economic dependence on psychological development during emerging adulthood, revealing how patriarchal norms perpetuate abusive behaviors in Egyptian society. While women showed a broader deterioration in wellbeing, spanning life satisfaction and positive affect, the stronger abuse–negative affect association among men indicates that both genders carry distinct vulnerabilities. The results emphasize the urgent need for culturally sensitive interventions that address both familial dynamics and gender-based vulnerabilities, while promoting the psychological wellbeing of emerging adults in Egypt and similar sociocultural contexts.

## 1 Introduction

The transition to adulthood represents a critical developmental period characterized by profound identity exploration, systemic instability, feeling in-between, and gradual negotiation of individual independence (Arnett, 2007). In Egypt, this transition has gained renewed significance following the reduction of the legal age of adulthood from 21 to 18 years (Constitution of the Arab Republic of Egypt, 2014), creating a substantial demographic of emerging adults (ages 18–29) that constitutes 21% of the population (Dhillon et al., 2009). Egypt's complex socio-cultural landscape—shaped by intersecting cultural traditions, economic constraints, and family dynamics—creates a distinct context where the psychological development of emerging adults faces significant challenges. Parental relationships are particularly pivotal, as they can either facilitate or hinder the achievement of key developmental milestones, such as autonomy, self-definition, and individual agency (Benson et al., 2011; Liu and Rahman, 2022).

The prolonged economic dependence of Egyptian emerging adults on their families is a crucial factor shaping their developmental trajectory (Assaad et al., 2022; Barsoum et al., 2014; Roushdy and Sieverding, 2015). With youth unemployment reaching 19.7% and a stark gender difference in labor market participation—16% women vs. 84% men (Deng et al., 2024; Ministry of Planning and Economic Development, 2023)—many young adults remain embedded within family systems well beyond legal adulthood. This extended dependence aligns with family systems theory, where individual development is deeply intertwined with family dynamics and intergenerational patterns (Bowen, 1978). Within Egypt's patriarchal family structures (Aref, 2020; Fishere, 2021; Madi et al., 2024), this dependence often sustains traditional disciplinary practices that can escalate into abusive behaviors, particularly under conditions of economic stress (Maly et al., 2023). While extensive research has documented the high prevalence of violent disciplinary practices in Egypt (Madi et al., 2024; Ministry of Health and Population et al., 2015; UNICEF, 2017a,b; Elghossain et al., 2019; Youssef et al., 1998; Abdeen et al., 2023), it is suggested that these culturally and economically reinforced behaviors may well persist into emerging adulthood.

The normalization of parental abuse can be understood through an ecological systems lens, which illuminates the interplay between individual development, family dynamics, and broader socio-cultural environments (Bronfenbrenner, 1979). At the macrosystem level, patriarchal norms and economic instability create conditions where abusive behaviors may be overlooked or justified. Although research predominantly focuses on childhood, larger-scale studies reveal that 42.3% of 28,175 participants experienced severe physical punishment (Abdel-Fatah, 2021), mirroring broader regional patterns where over 106 million children in the Middle East and North Africa (MENA) region regularly face violent discipline at home (UNICEF, 2018). Cultural values emphasizing family honor and social cohesion contribute to systematic underreporting, particularly where protecting family reputation supersedes individual wellbeing (Elghossain et al., 2019). This is further complicated by parental reluctance to acknowledge harmful behaviors, children's fear of retaliation, and feelings of shame or guilt (Ali et al., 2024; Madi et al., 2024).

Understanding these patterns requires clarity about what constitutes abuse vs. maltreatment. While the terms are often used interchangeably, they carry distinct meanings. “Maltreatment” serves as an umbrella term encompassing various forms of harm, including neglect, whereas “abuse” refers specifically to intentional acts of harm inflicted directly on a child—physical, psychological, or sexual in nature—as defined in DSM-5 and ICD-10 (Slep et al., 2015). According to the World Health Organization (World Health Organization, 2022a), abuse constitutes any non-accidental physical and emotional ill-treatment, sexual abuse, or exploitation directed at a child, leading to potential or actual harm to health, survival, development, or dignity, typically perpetrated within relationships of responsibility, trust, or power. A further distinction concerns “harsh discipline,” which refers to punitive parental behaviors—such as corporal punishment or psychological coercion—that may be culturally framed as corrective rather than harmful. Critically, the developmental stage of the recipient matters: behaviors framed as discipline during adolescence become developmentally inappropriate when directed at adults and should therefore be reconceptualized as abuse. This reconceptualization reflects the expectation that parental roles shift from control and discipline toward guidance and support during emerging adulthood (Jablonski and Martino, 2013; McKinney et al., 2022; McMahon, 2014).

In this study, perceived parental abuse is operationalized as emerging adults' subjective experiences of parental behaviors perceived as intentional, non-accidental, and harmful. These behaviors are conceptualized across two empirically derived dimensions: Overt Abuse (physical acts such as hitting, slapping; verbal acts such as name-calling, insults) and Covert Abuse (relational acts such as exclusion, emotional blackmail, spreading false information, manipulating social situations), all perpetrated within relationships characterized by trust, responsibility, and power asymmetry. This operationalization is deliberately delimited in two important respects. First, sexual abuse is excluded. Although it constitutes a recognized and severe form of abuse within both DSM-5 and ICD-10, it represents a clinically distinct category requiring dedicated measurement instruments and separate analytical treatment. Second, unlike harsh discipline—which retains a corrective intent however inappropriately—the behaviors captured by the Perceived Parental Abusive Behaviors Scale (PPABS), a self-report questionnaire developed for this study, exceed that corrective threshold as perceived by the recipient. When such behaviors are directed at emerging adults—a developmental stage distinct from childhood—they are reconceptualized as abuse, as the parent-child relationship is no longer defined by unilateral authority over discipline. Thus, a higher score on the PPABS indicates greater perceived exposure to parental abuse as defined herein, as measured by the Overt Abuse and Covert Abuse subscales of the PPABS.

Emerging adulthood is a developmentally distinct stage that differs from adolescence in ways that fundamentally alter parent–child dynamics. First, autonomy development becomes a central task, with young people striving for functional and emotional independence while often remaining dependent on parents for financial support, housing, and emotional support (Cohen et al., 2003; Maly et al., 2023). (Biggart and Walther 2006) document the “yo-yo transitions” characteristic of this period, wherein many individuals return to more dependent roles even after initially assuming adult responsibilities. Second, this continued dependence creates unique vulnerabilities: emerging adults who rely on parents for material or emotional support may lack the practical means to exit abusive family situations (Jablonski and Martino, 2013). Third, identity exploration and role transitions across education, work, and relationships create both opportunities for positive change away from maladaptive family dynamics and heightened susceptibility to existing vulnerabilities (Williams, 2016). Taken together, these characteristics necessitate a fundamental renegotiation of parent–child relationships, with expectations shifting from parental authority toward guidance, advice, and mutual respect (Jablonski and Martino, 2013).

Gender dynamics are associated with the experience and reporting of parental abuse (World Health Organization, 2022b). Egypt's ranking of 116 out of 189 countries on the Gender Inequality Index (UNFPA Egypt, 2019) reveals the broader societal differences that manifest within family structures. Current data of women aged 15–45 indicates concerning patterns: 50.2% of married women experience spousal violence—including psychological abuse (49.5%), restrictions on liberties (19.7%), physical violence (16.1%), and sexual violence (13.8%)—while 57% face domestic non-spousal violence (Rashed and Allam, 2022). This aligns with a documented 19% rise in domestic violence in Egypt (National Council for Women, 2021), affecting approximately 7.8 million women, mostly within family settings (Duvvury et al., 2015). These patterns align with gender role socialization theory (Eagly, 1987), which illustrates how traditional gender norms create distinct vulnerabilities for both men and women within patriarchal societies (Human Rights Watch, 2024). Women, socialized to prioritize obedience and familial harmony, often encounter increasing risk of psychological and emotional abuse. Meanwhile, men face pressures to conform to rigid masculinity notions, leading to different forms of familial conflict. Studies indicate differentiated patterns of abuse: boys more frequently encounter physical violence, while girls face elevated rates of psychological and sexual abuse (Abdel-Fatah, 2021). However, Egypt lacks population-based statistics and reliable official records on parental abuse, particularly concerning emerging adults. The absence of disaggregated data on non-spousal domestic violence from parents further compounds this knowledge gap, limiting our understanding of how gendered patterns of abuse evolve across developmental stages.

Furthermore, multiple complementary theoretical frameworks support that gender moderates the relationship between parental abuse and subjective wellbeing. Gender role socialization theory (Eagly, 1987) posits that societal expectations prescribe distinct psychological dispositions and behavioral repertoires for men and women. In patriarchal societies such as Egypt, these processes are particularly pronounced: women are socialized toward emotional expressiveness, relational orientation, and familial obedience, while men are socialized toward emotional restraint, assertiveness, and conformity to rigid masculinity norms (Shousha, 2020). These divergent socialization pathways create gender-specific vulnerabilities in response to parental abuse. Women are more likely to internalize abuse experiences—manifesting depression, anxiety, rumination, and self-blame—whereas men are more likely to externalize distress through aggression and risk-taking behavior (Maschi et al., 2008; Topitzes et al., 2011).

The correlates of parental abuse during emerging adulthood are evident across multiple dimensions of wellbeing, understood through self-determination theory's emphasis on autonomy, competence, and relatedness as fundamental psychological needs (Ryan and Deci, 2000). Parental abuse—encompassing overt (physical and verbal) and covert (emotional and relational) forms—is associated with how individuals feel, function, and evaluate their lives (Jarden and Roache, 2023). When basic psychological needs are thwarted in abusive family environments, emerging adults experience diminished subjective wellbeing, a core component of mental health (World Health Organization, 2022c) and flourishing across life domains (Lee et al., 2021; Lhaksampa et al., 2024). Within Egyptian contexts, though research on emerging adults remains limited, available studies document decreased confidence and psychological security (Gao et al., 2024; Hedeia, 2018), alongside increased negative affect and various psychological disorders (Abo-Al-Ata and Ead, 2019), suggesting that the association between parental abuse and wellbeing may be particularly pronounced during this developmental period.

Subjective wellbeing, mentioned above as the outcome through which these effects are registered, is conceptualized in the present study as a tripartite construct comprising one cognitive and two affective components (Diener, 1984; Diener et al., 1985; Watson et al., 1988). The cognitive component, life satisfaction, is a global and relatively stable evaluative judgment in which individuals assess the quality of their lives as a whole against their own standards rather than against externally imposed criteria. The affective components are positive affect, the frequency with which pleasant emotional states such as interest, enthusiasm, and joy are experienced, and negative affect, the frequency of aversive states such as anger, distress, and anxiety. Critically, positive and negative affect are not opposite ends of a single continuum but semi-independent dimensions with partially distinct determinants and correlates (Watson et al., 1988). An adverse circumstance may therefore deplete the frequency of pleasant emotion without elevating unpleasant emotion or elevate distress while leaving evaluative judgments of life as a whole intact. High subjective wellbeing is accordingly defined by the conjunction of high life satisfaction, frequent positive affect, and infrequent negative affect, and each component must be examined in its own right. Because these components may respond differently to the same family environment, the present study specifies its hypotheses separately for each.

Despite extensive research documenting the effects of childhood abuse on adult wellbeing, significant methodological and contextual gaps persist. Current studies predominantly rely on retrospective approaches (e.g., Abo-Al-Ata and Ead, 2019, 2020; Fishere and Habermas, 2023; Hedeia, 2018; Ismail and Atar, 2018; Kotb, 2012), which face challenges of memory reconstruction and recall bias (Reuben et al., 2016; Scott et al., 2012). In Egypt specifically, research focuses heavily on children under 18 (UNICEF, 2015), while data on emerging adults remains limited and largely retrospective. This gap is particularly pronounced given Egypt's unique sociocultural context. The present study addresses these limitations by examining the current prevalence of perceived parental abuse among Egyptian emerging adults—that is, abuse reported as ongoing in the present parent–child relationship rather than recalled from childhood—exploring gender-specific experiences and investigating the relationship between current abuse and subjective wellbeing. Hence, the research hypotheses can be formulated as follows:

*H1: Emerging adults will report experiencing some form of parental abuse*.

*H2: Emerging adult women will report higher rates of experiencing parental abusive behaviors compared to emerging adult men*.

*H3a: Higher levels of perceived parental abuse will be associated with lower life satisfaction*.

*H3b: Higher levels of perceived parental abuse will be associated with lower positive affect*.

*H3c: Higher levels of perceived parental abuse will be associated with higher negative affect*.

*H4a: Gender will moderate the abuse–life satisfaction relationship, such that the negative association will be stronger for emerging adult women than men*.

*H4b: Gender will moderate the abuse–positive affect relationship, such that the negative association will be stronger for emerging adult women than men*.

*H4c: Gender will moderate the abuse–negative affect relationship, such that the positive association will be stronger for emerging adult women than men*.

## 2 Methods and materials

### 2.1 Participants and data collection

A cross-sectional design was adopted to examine the moderating role of gender in the relationship between perceived abusive parental behaviors and subjective wellbeing. A convenience sample of 360 early emerging Egyptian adults (121 men, 239 women) aged 18–21 years (*M* = 19.43, *SD* = 1.07) was recruited from three universities: two public institutions and one private institution. The age range of 18–21 was selected to capture the earliest phase of emerging adulthood, during which legal adulthood has recently been attained but economic and residential dependence on parents remains near-universal in the Egyptian context, creating specific vulnerability to ongoing parental abuse.

Participant characteristics are presented in Table 1. As a university-based convenience sample, the findings provide insights into this specific demographic but are not generalisable to non-student emerging adults in Egypt. Prior to data collection, formal ethical approval was obtained from the Institutional Research Ethics Committee at Cairo University; the two remaining participating institutions accepted this approval for the purposes of the present study. A mixed-mode strategy was adopted to maximize accessibility and response rates. Module leaders at the three participating universities were contacted and invited to allow data collection during their scheduled classes. In classes where permission was granted, the researchers attended in person to administer the survey. The format used in each class was determined by the logistics of the session, including classroom facilities and the module leader's preference. Where the online format was used, a QR code linking to the survey was shared with the class through the relevant student cohort university mailing list, class WhatsApp group, or other class communication platform; the form was configured to accept a single response per respondent. Where the paper-and-pencil format was used, questionnaires were distributed and collected during the session. The researchers were present throughout all sessions to ensure standardized administration and to answer participants' questions.

**Table 1:** Characteristics of early emerging adult participants.

Participants' characteristic	*n* men	%	*n* women	%	*n* full	%
Gender	121	33.6	239	66.4	360	100
Living arrangements						
Temporarily away for studying	13	10.7	40	16.7	53	14.7
Living with parents	108	89.3	199	83.3	307	85.3
Financial dependence on parents						
Completely dependent	98	81.0	230	96.2	328	91.1
Mostly dependent	19	15.7	9	3.8	28	7.8
Somewhat dependent	3	2.5	0	0	3	0.8
Mostly independent	1	0.8	0	0	1	0.3
Sibship						
Having siblings	119	98.3	237	99.2	356	98.9
Being an only child	2	1.7	2	0.8	4	1.1
Birth order						
1st child	62	51.2	86	36.0	148	41.1
2nd child	44	36.4	75	31.4	119	33.1
3rd child or later	15	12.4	78	32.6	93	25.8
Major specialization						
Natural sciences	14	11.6	8	3.3	22	6.1
Social sciences and humanities	107	88.4	231	96.7	338	93.9

Upon accessing the survey, all participants were provided with a detailed information sheet outlining the study's purpose, procedures, and the measures in place to protect their confidentiality. Informed consent was obtained without written signature. Online participants consented electronically by selecting an agreement option. Paper-and-pencil participants consented verbally and by ticking an agreement box on the questionnaire. All participants were informed of their right to withdraw at any time without penalty. The Institutional Research Ethics Committee at Cairo University waived written signature requirements given the low-risk nature of the study and to protect participant anonymity. The survey took approximately 30 min to complete. Upon completion, participants were debriefed and provided with a list of mental health resources to contact if needed.

### 2.2 Measures

#### 2.2.1 Perceived parental abusive behaviors scale (PPABS)

This scale was developed by the authors to assess early emerging adults' current perceptions of parental abusive behaviors, as experienced in their present stage of life. Participants rated each item based on their current, ongoing experiences with their parents, reflecting present relationship dynamics rather than retrospectively recalling experiences from a past specific time frame. The instrument is a self-report questionnaire administered at a single time point; it therefore assesses recent and ongoing experience as perceived by the respondent and does not constitute momentary or event-contingent assessment. Unlike existing tools that primarily focus on retrospectively measuring childhood experiences of parental abuse—such as those used in Egyptian rural governorates (Abo-Al-Ata and Ead, 2019, 2020; Fishere and Habermas, 2023; Hedeia, 2018; Ismail and Atar, 2018; Kotb, 2012) and other Arab regional contexts, including studies in the Kingdom of Saudi Arabia (Al-Mosahar, 2007) and Yemen (Al-Katheri, 2023)—this scale is specifically designed to capture currently ongoing experiences of abuse as perceived by emerging adults in the Egyptian context. This focus is critical because emerging adults may perceive and respond to parental abuse differently due to their developmental stage, increased autonomy, and evolving relationships with their parents.

The development process included a thorough review of existing instruments, expert consultations, and a pilot study with 87 participants, recruited independently from the main study sample. Based on qualitative insights from the pilot study, a 37-item questionnaire was created, measuring Overt (physical and verbal) and Covert (emotional and relational) abuse on a 5-point Likert scale (1 = Never Happens, 5 = Happens All the Time). Content validity was assessed by a panel of six academic experts, who evaluated the necessity, relevance, and clarity of the items. All items were retained, indicating satisfactory content validity.

Principal components analysis (PCA) with varimax rotation was then conducted to examine the underlying structure of the scale. This analysis revealed a two-component structure. The first component, Overt Abuse (26 items), accounted for 49.26% of the variance (eigenvalue = 18.23). It encompassed physical and verbal abusive behaviors such as hitting, kicking, slapping, name-calling, insults, teasing, intimidation, and the use of humiliating nicknames. Representative items with high loadings included: “I get hit by both my parents or one of them when we argue” (loading = 0.78), “Both my parents or one of them mock my way of talking and the tone of my voice” (loading = 0.85), and “My parents or one of them curse at me for trivial reasons” (loading = 0.76).

The second component, Covert Abuse (nine items), accounted for an additional 5.07% of the variance (eigenvalue = 1.87). It captured emotional and relational manipulative behaviors, such as exclusion, spreading rumors, emotional blackmailing, and manipulating social situations. Representative high-loading items included: “My parents threaten to stop supporting me if I don't do what they want” (loading = 0.72), “They spread false information about me to others” (loading = 0.93), and “My parents or one of them belittle my abilities and potential in front of others” (loading = 0.80).

Following PCA, the scale was reduced from 37 to 35 retained items. The combined two-component solution explained 54.33% of the total variance, with all items demonstrating significant loadings above the 0.40 threshold, indicating a well-defined structure (Fornell and Larcker, 1981; Hair et al., 2019).

Construct validity was further supported by composite reliability (CR) and average variance extracted (AVE). CR values ranged from 0.90 to 0.96, and AVE values ranged from 0.50 to 0.52, supporting convergent validity (Cheung et al., 2024; Fornell and Larcker, 1981; Hair et al., 2019). The heterotrait-monotrait ratio (HTMT) was 0.87, indicating that the two components were related but empirically distinguishable (Henseler et al., 2015).

Confirmatory factor analysis (CFA) was conducted on the main study sample (*N* = 360), which was independent of the pilot sample (*n* = 87). The factor loadings ranged from 0.56 to 0.92 for the first factor, Overt Abuse, and from 0.54 to 0.89 for the second factor, Covert Abuse. All item loadings exceeded the recommended threshold of 0.50 (Hair et al., 2019), indicating satisfactory item–factor relationships and providing support for the proposed two-factor structure. Furthermore, all factor loadings were significant (*p* < 0.001). The model fit indices demonstrated acceptable overall fit to the data (Hu and Bentler, 1999), with CFI = 0.92, TLI = 0.90, SRMR = 0.06, RMSEA = 0.07. Collectively, these findings support the adequacy of the two-factor measurement model.

The PPABS also demonstrated strong internal consistency. Cronbach's alpha was 0.97 for the overall scale, 0.96 for the Overt Abuse subscale, and 0.88 for the Covert Abuse subscale. McDonald's omega was 0.95 for the total scale, 0.95 for Overt Abuse, and 0.89 for Covert Abuse (McDonald, 1999). The average inter-item correlation was 0.48 for the overall scale, falling within the recommended range of 0.15–0.50 (Clark and Watson, 1995), indicating acceptable item overlap without redundancy. The higher overall alpha compared to the subscales partly reflects the larger number of items in the full scale (Cortina, 1993). Overall, the PPABS showed strong psychometric properties and appears to be a reliable and valid measure of perceived parental abusive behaviors among early emerging adults.

#### 2.2.2 Subjective wellbeing questionnaire

Subjective wellbeing was assessed using Mohammed's (2009) Arabic adaptation of both Diener et al.'s (1985) Satisfaction with Life Scale and Watson et al.'s (1988) Positive and Negative Affect Scale. The Satisfaction with Life Scale consists of 5 items measuring cognitive appraisal of life satisfaction (e.g., “I am satisfied with my life”), rated on a 5-point scale (1 = Strongly Disagree, 5 = Strongly Agree). The Positive and Negative Affect Scale includes 12 items (six positive, six negative) measuring affective components of wellbeing (e.g., “joy,” “anger”), rated on a 5-point scale (1 = Never, 5 = Always). Both subscales—of the Subjective Wellbeing Questionnaire—Diener et al.'s (1985) Satisfaction with Life Scale and Watson et al.'s (1988) Positive and Negative Affect Scale—have demonstrated high reliability and validity in previous studies. These subscales showed good to excellent internal consistency, with Cronbach's alpha coefficients of 0.78 for the Satisfaction with Life subscale, 0.87 for the Positive Affect subscale, and 0.84 for the Negative Affect subscale.

## 3 Results

### 3.1 Prevalence of the perceived parental abusive behaviors (H1)

Descriptive analyses were used to examine the prevalence of perceived parental abusive behaviors among the participants, distinguishing between emerging adult men and women. For descriptive purposes, responses on the Perceived Parental Abusive Behaviors Scale were categorized into low, moderate, and high levels of abuse using visual binning in SPSS. The cut-off points were determined empirically based on the score distribution of the current sample (Kotsiantis and Kanellopoulos, 2006). This categorization was used solely to facilitate interpretation and description of abuse levels in Table 2. All inferential statistical analyses (*t*-tests, correlations, regressions) were conducted using the original continuous scores. As shown in Table 2, 63.6% of participants reported experiencing some form of parental abuse, with 32.8% reporting high levels and 30.8% reporting moderate levels. When categorized by type, overt abuse (physical and verbal) was reported by 65.5% of participants (32.2% high, 33.3% moderate), while covert abuse (emotional and relational) was reported by 56.4% (33.3% high, 23.1% moderate). These findings support H1: a majority of early emerging adults reported currently experiencing moderate-to-high levels of parental abuse.

**Table 2:** Prevalence of perceived parental abuse among early emerging adults.

Prevalence level	*f* all *N* = 360	%	*f* men *N* = 121	%	*f* women *N* = 239	%
Perceived parental abusive behaviors (total)						
Low	131	36.4	68	56.2	63	26.4
Moderate	111	30.8	25	20.7	86	36.0
High	118	32.8	28	23.1	90	37.7
Overt abuse (physical and verbal)						
Low	124	34.4	70	57.9	54	22.6
Moderate	120	33.3	23	19.0	97	40.6
High	116	32.2	28	23.1	88	36.8
Covert abuse (emotional and relational)						
Low	157	43.6	66	54.5	91	38.1
Moderate	83	23.1	24	19.8	59	24.7
High	120	33.3	31	25.6	89	37.2

### 3.2 Gender differences in perceived parental abuse (H2)

Supporting H2, emerging adult women aged 18–21 years reported significantly higher rates of perceived parental abuse compared to early emerging adult men. Specifically, 73.7% of women reported experiencing abuse, compared to 43.8% of men. This pattern was consistent across both overt abuse (77.4% women vs. 42.1% men) and covert abuse (61.9% women vs. 45.4% men). Independent samples *t*-tests confirmed these differences as significant for the total scale and for both subtypes, with women scoring higher in every comparison (Table 3). Although gender differences were significant and consistently favored women, effect sizes were uniformly small, indicating that the magnitude of these differences was modest in practical terms.

**Table 3:** Gender differences in perceived parental abusive behaviors.

Variable	*M* men	*SD*	*M* women	*SD*	*t*(358)	*p*	Cohen's *d*
Perceived parental abuse	48.74	20.08	55.87	21.69	3.02	0.003	0.34
Overt abuse	37.18	15.92	43.13	17.67	3.12	0.002	0.35
Covert abuse	11.56	4.54	12.74	4.96	2.19	0.029	0.24

### 3.3 Relationship between perceived parental abuse and subjective wellbeing (H3a–H3c)

Pearson's correlation coefficients were used to test H3a–H3c (Table 4). Contrary to H3a, perceived parental abuse was not significantly correlated with life satisfaction, *r* = −0.06, *p* = 0.280. Supporting H3b, perceived parental abuse was significantly and negatively associated with positive affect. H3c was also supported as perceived parental abuse was significantly and positively associated with negative affect, the strongest association observed. In terms of magnitude, associations ranged from small to medium according to Cohen's (1988) benchmarks.

**Table 4:** Pearson's correlation coefficients between perceived parental abusive behaviors and early emerging adults' subjective wellbeing.

Variable	Satisfaction with life	Positive affect	Negative affect
Perceived parental abuse	−0.06	−0.22^***^	0.46^***^
Overt abuse	−0.07	−0.23^***^	0.46^***^
Covert abuse	0.01	−0.14^**^	0.39^***^

*N* = 360. ^**^p < 0.01. ^***^p < 0.001.

The two abuse subtypes followed the same pattern as the total scale score. Neither overt nor covert abuse was associated with life satisfaction, whereas both were negatively associated with positive affect and positively associated with negative affect. Taken together, the affective components of subjective wellbeing—and negative affect in particular—proved more sensitive to perceived parental abuse than the cognitive-evaluative component. As shown in Section 3.5, the null bivariate association between abuse and life satisfaction reflected opposing gender-specific associations that canceled each other out at the whole-sample level.

### 3.4 Demographic correlates of the study variables

Prior to the moderation analyses, Pearson correlations were computed between the demographic variables (gender, age, major specialization, birth order, and living arrangement) and the main study variables (Table 5). Sibship and financial dependence were excluded from these analyses because they were near-constant in this sample, precluding meaningful correlation estimates. Gender was significantly associated with perceived parental abuse and with both affective components of wellbeing, but not with life satisfaction: women reported more abuse, lower positive affect, and higher negative affect than men. Age showed the same pattern across the same three variables, with older participants within this narrow band reporting more abuse, more negative affect, and less positive affect. Major specialization and living arrangement were each associated with life satisfaction alone, students living with their parents reporting slightly lower life satisfaction. Birth order was unrelated to every study variable. All significant demographic associations were small in magnitude, the largest being that between age and negative affect. Accordingly, age, major specialization, and living arrangement were retained as covariates in all moderation models; gender was already included as the moderator.

**Table 5:** Correlations between demographic variables and main study variables (*N* = 360).

Demographic	Abuse	Life satisfaction	Positive affect	Negative affect
Gender	−0.16^**^	0.02	0.12^*^	−0.15^**^
Age	0.15^**^	−0.05	−0.13^**^	0.18^***^
Major specialization	−0.02	0.11^*^	0.02	−0.02
Birth order	−0.01	−0.04	−0.09	−0.03
Living arrangement	0.04	−0.12^*^	−0.08	0.08

*N* = 360. Gender coded 0 = women, 1 = men. Living arrangement coded 1 = temporarily away for studying, 2 = living with parents. Major specialization coded 1 = social sciences and humanities, 2 = natural sciences. Age is reported in years as a continuous variable. Birth order is ordinal (1 = first child, 2 = second child, 3 = third child or later). Sibship and financial dependence were excluded because both were near-constant in this sample (1.1% only children; 91.1% completely financially dependent). ^*^p < 0.05. ^*^^*^p < 0.01. ^*^^*^^*^p < 0.001.

### 3.5 Moderating role of gender (H4a–H4c)

Moderation analyses were conducted using the PROCESS macro for SPSS (Model 1; Hayes, 2022). PROCESS was chosen because it estimates the conditional effect of the predictor at each level of the moderator with significance tests and 95% confidence intervals, tests the interaction as a change in *R*^2^, and generates the data needed for interaction plots. In each model, perceived parental abuse (mean-centered) predicted one dimension of subjective wellbeing, with gender (0 = women, 1 = men) as moderator and age, major specialization (1 = social sciences and humanities, 2 = natural sciences), and living arrangement (1 = temporarily away for studying, 2 = living with parents) as covariates (see Section 3.4). Results are summarized in Table 6 and Figures 1–3. Prior to analysis, regression assumptions were examined. Residuals were approximately normally distributed and homoscedastic (*M* = 0.00, *SD* = 0.99). Multicollinearity was not a concern (VIF ≤ 1.52; Hair et al., 2019), and no influential cases were identified (max Cook's distance = 0.05).

**Table 6:** Process model 1 results: conditional effects of perceived parental abuse on wellbeing by gender, controlling for age, major specialization, and living arrangement (*N* = 360).

Outcome/effect	*B*	*SE*	*t*	*p*	95% CI
Life satisfaction (*R*^2^ = 0.10, *F*_(6, 353)_ = 6.36, *p*<0.001)					
Abuse × gender (Δ*R*^2^ = 0.07)	0.12	0.02	5.15	< 0.001	[0.07, 0.16]
Conditional effect: women	−0.05	0.01	−3.68	< 0.001	[−0.07, −0.02]
Conditional effect: men	0.07	0.02	3.71	< 0.001	[0.03, 0.11]
Positive affect (*R*^2^ = 0.08, *F*_(6, 353)_ = 5.01, *p*<0.001)					
Abuse × gender (Δ*R*^2^ = 0.01)	0.05	0.02	2.14	0.033	[0.00, 0.10]
Conditional effect: women	−0.06	0.01	−4.26	< 0.001	[−0.08, −0.03]
Conditional effect: men	−0.01	0.02	−0.30	0.761	[−0.05, 0.03]
Negative affect (*R*^2^ = 0.25, *F*_(6, 353)_ = 19.06, *p*<0.001)					
Abuse × Gender (Δ*R*^2^ *=* 0.02)	0.07	0.02	2.76	0.006	[0.02, 0.11]
Conditional effect: women	0.08	0.01	6.03	< 0.001	[0.05, 0.11]
Conditional effect: men	0.15	0.02	7.39	< 0.001	[0.11, 0.19]

B = unstandardized coefficient; conditional effects are simple slopes of abuse within each gender. All continuous predictors were mean-centered. Gender coded 0 = women, 1 = men. Age, major specialization, and living arrangement were entered as covariates. Values rounded to two decimal places. For positive affect, the interaction lower bound is 0.004 (displayed as 0.00); the CI excludes zero.

**Figure 1:**
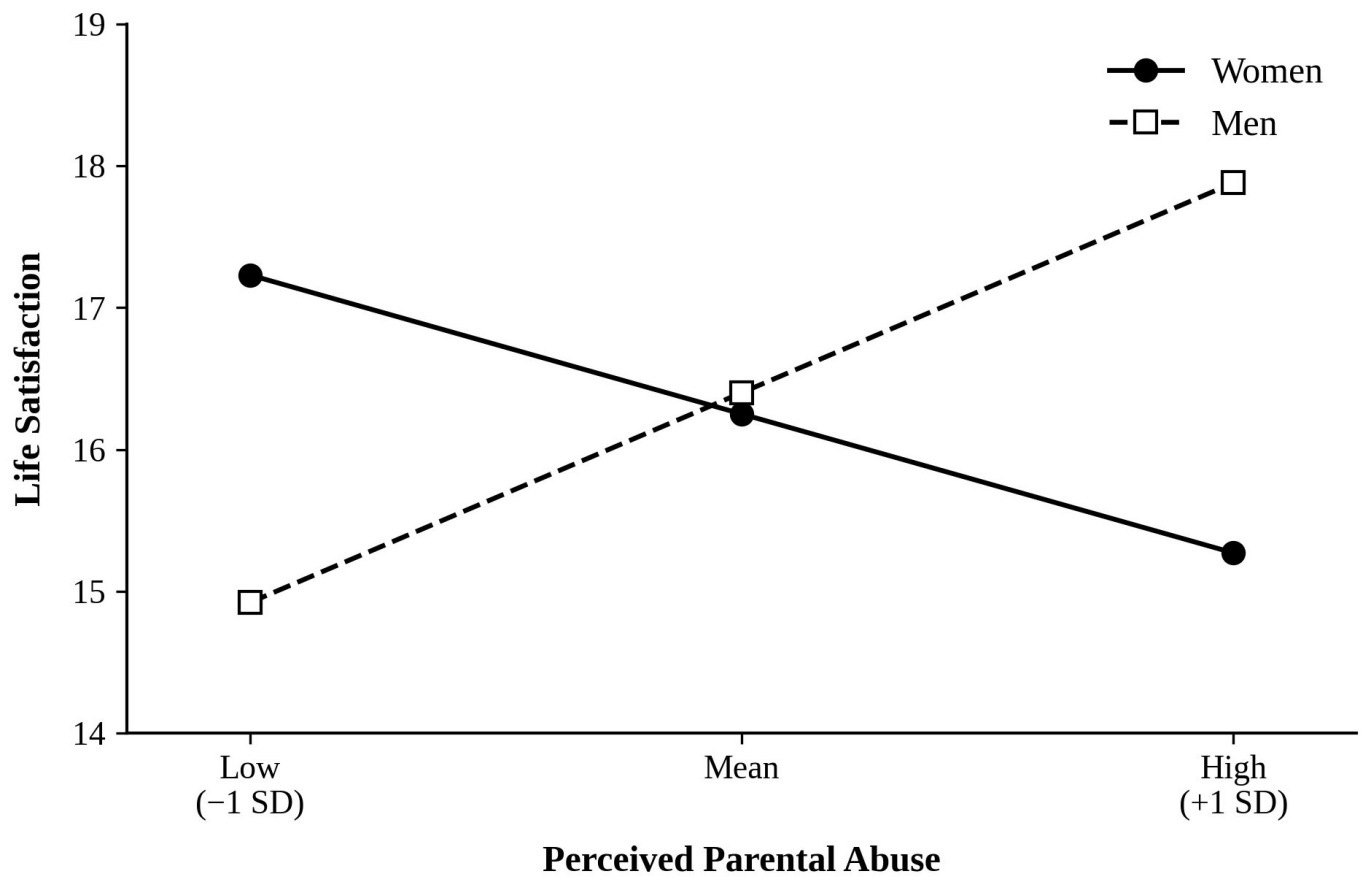
Gender moderates the association between perceived parental abuse and life satisfaction.

**Figure 2:**
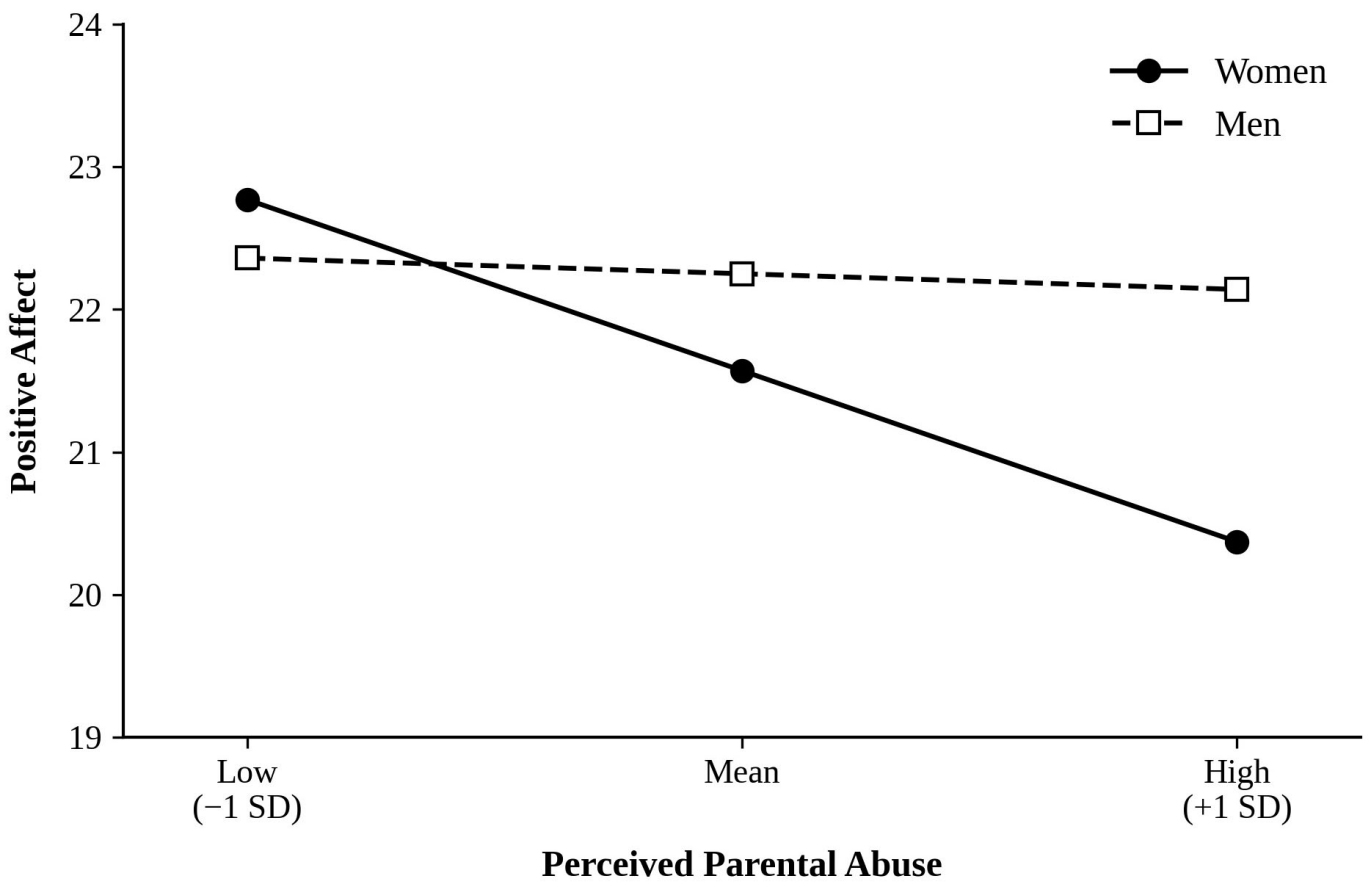
Gender moderates the association between perceived parental abuse and positive affect.

**Figure 3:**
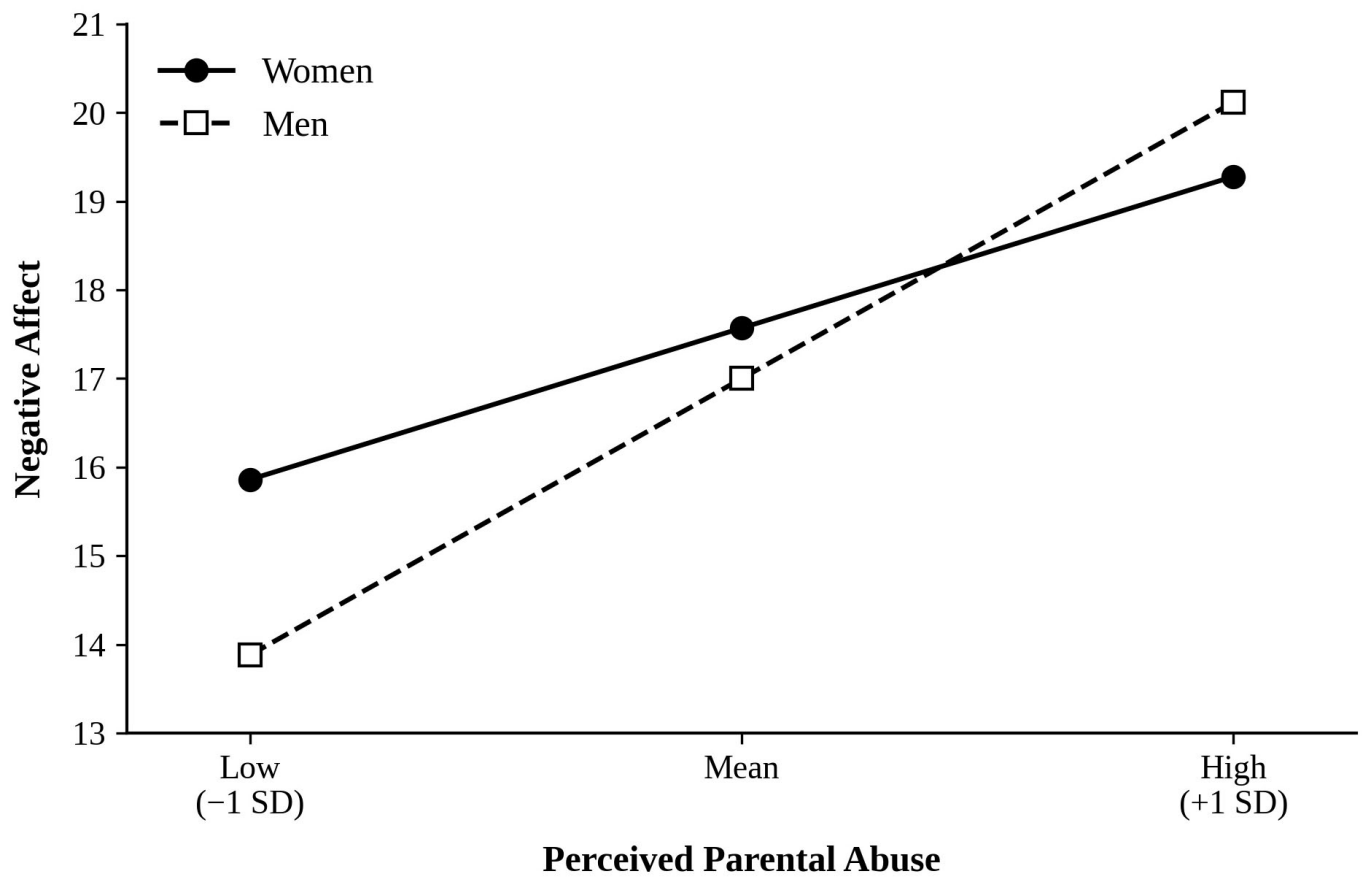
Gender moderates the association between perceived parental abuse and negative affect.

For life satisfaction (H4a), the abuse × gender interaction was significant, *B* = 0.12, Δ*R*^2^ = 0.07. Conditional effects showed that higher perceived abuse was associated with significantly lower life satisfaction among women, *B* = −0.05, whereas among men the association was, unexpectedly, significantly positive, *B* = 0.07 (Figure 1). H4a was therefore partially supported: the predicted negative association emerged for women and was significantly stronger (more negative) than for men, but the significant positive association among men was not anticipated and constitutes a crossover interaction.

For positive affect (H4b), the interaction was significant, *B* = 0.05, Δ*R*^2^ = 0.01. As predicted, higher abuse was associated with significantly lower positive affect among women, *B* = −0.06, whereas the association was non-significant among men, *B* = −0.01 (Figure 2). H4b was supported: the detrimental association between perceived parental abuse and positive affect was evident among women but absent among men.

For negative affect (H4c), the interaction was significant, *B* = 0.07, Δ*R*^2^ = 0.02. Higher abuse was associated with significantly higher negative affect in both groups; however, contrary to H4c, the association was stronger among men, *B* = 0.15, than among women, *B* = 0.08 (Figure 3). H4c was therefore not supported in the predicted direction, although gender did condition the strength of the abuse–negative affect association. Across all three models, age, major specialization, and living arrangement were controlled; the hypothesized interactions thus held independently of these demographic influences.

## 4 Discussion

The findings of this study provide empirical evidence on the concerning prevalence of parent-perpetrated abuse experienced by early emerging adults and its association with diminished subjective wellbeing. While previous literature has often focused on the cumulative effects of child abuse on adolescents and adults through retrospective reports, this study highlights the significant prevalence of reported parental abuse among early emerging adults as they experience it. H1 was supported: in this study, 63.6% of the participants reported moderate to high levels of abuse occurring in early emerging adulthood aged 18–21 years, with 65.5% experiencing overt (physical and verbal) abuse and 56.4% experiencing covert (emotional and relational) abuse. These findings align with studies investigating parental-perpetrated abuse prevalence among college students in Italy (Maly et al., 2023), the Middle Eastern region (Elsayed et al., 2022) and Southern USA (McKinney et al., 2020), suggesting that parental abuse during the early phase of emerging adulthood is a global issue with culturally specific manifestations. These high rates of abuse can be attributed to several socio-cultural and economic factors within Egyptian society. Physical punishment is widely normalized as a parenting tool, reflecting cultural values that prioritize strict obedience and hierarchical family structures (Abdel-Fatah, 2021; Madi et al., 2024; Youssef et al., 1998). Additionally, Egypt's economic challenges—characterized by high unemployment and poverty rates—create immense pressure on families, exacerbating tensions and increasing the likelihood of abusive behaviors (Fishere, 2021; Lewis and Werr, 2023; Potkanski-Palkam, 2024). Emerging adults are particularly embedded within these economic struggles through a complex dependency cycle. While marriage traditionally serves as a culturally sanctioned pathway to independence, rising costs and financial instability have significantly delayed the average marriage age (Crocetti and Meeus, 2014; Jyrkiäinen and Bisset, 2016). This delay, combined with high youth unemployment and housing costs (Ministry of Planning and Economic Development, 2023), necessitates extended family co-residence. While such living arrangements align with cultural emphasis on “familism” and can provide financial support (Scabini et al., 2006), they may also increase vulnerability to abuse, especially within patriarchal structures emphasizing control-oriented parenting (Madi et al., 2024; Maly et al., 2023). This prolonged dependence may significantly compromise emerging adults' development of autonomy and heighten their exposure to continued parental abuse.

In addition, H2 was supported: Egyptian early emerging adult women reported experiencing parental abuse at significantly higher rates than men. A striking 73.7% of emerging adult women reported experiencing some form of abuse, compared to 43.8% of men. This disparity is particularly evident in overt abuse (77.4% of early emerging adult women vs. 42.1% of men) and covert abuse (61.9% of early emerging adult women vs. 45.4% of men). Despite a limited amount of research on gender differences in parental abuse in non-western cultures (Xiao et al., 2023), these findings align with reports from the United Nations (Duvvury et al., 2015; National Council for Women, 2021) and the European Union (Rashed and Allam, 2022), which highlight the gender-based violence experienced by Egyptian women. The higher prevalence of abuse among early emerging adult women reflects broader societal inequalities and traditional gender roles that prioritize obedience and familial harmony for women, often at the expense of their autonomy and wellbeing (Eagly, 1987; Haj-Yahia, 2000).

Alternative explanations for these gender differences warrant consideration as well. The observed differences may partly reflect differential reporting tendencies rather than true prevalence differences. Men socialized within patriarchal norms emphasizing emotional stoicism and self-reliance may be less willing to disclose experiences of parental abuse, particularly psychological and relational forms, due to stigma and social expectations around masculinity (Courtenay, 2000). Additionally, women and men may have different thresholds for perceiving specific parental behaviors as abusive. Women socialized toward emotional expressiveness may be more attuned to psychological forms of abuse, whereas men may normalize certain harsh parental behaviors as consistent with expectations of male independence and toughness.

The results showed that gender moderated the association between perceived parental abuse and all three components of subjective wellbeing—but not uniformly, and not in the simple “stronger for women” pattern the hypotheses predicted. The moderation increment was substantial for life satisfaction (Δ*R*^2^ = 0.07) and more modest for the affective outcomes (Δ*R*^2^ = 0.01–0.02), yet the direction of the conditional effects, not merely their size, carries the theoretical weight. The full pattern can be interpreted through two complementary gendered dynamics operating within the Egyptian family system: *differential exposure*—who spends time inside the household, encounters its hostility, and who has access to compensatory life domains beyond it—and *differential expression*—which channel of wellbeing registers the harm, given the gender-specific socialization of emotional life into internalizing and externalizing response patterns (Eagly, 1987; Maschi et al., 2008; Shousha, 2020). On this account, women in this sample live the abuse relationally and comprehensively, whereas men escape parts of its evaluative reach while absorbing its emotional charge. Figure 4 summarizes this proposed model; each dimension is interpreted in turn.

**Figure 4:**
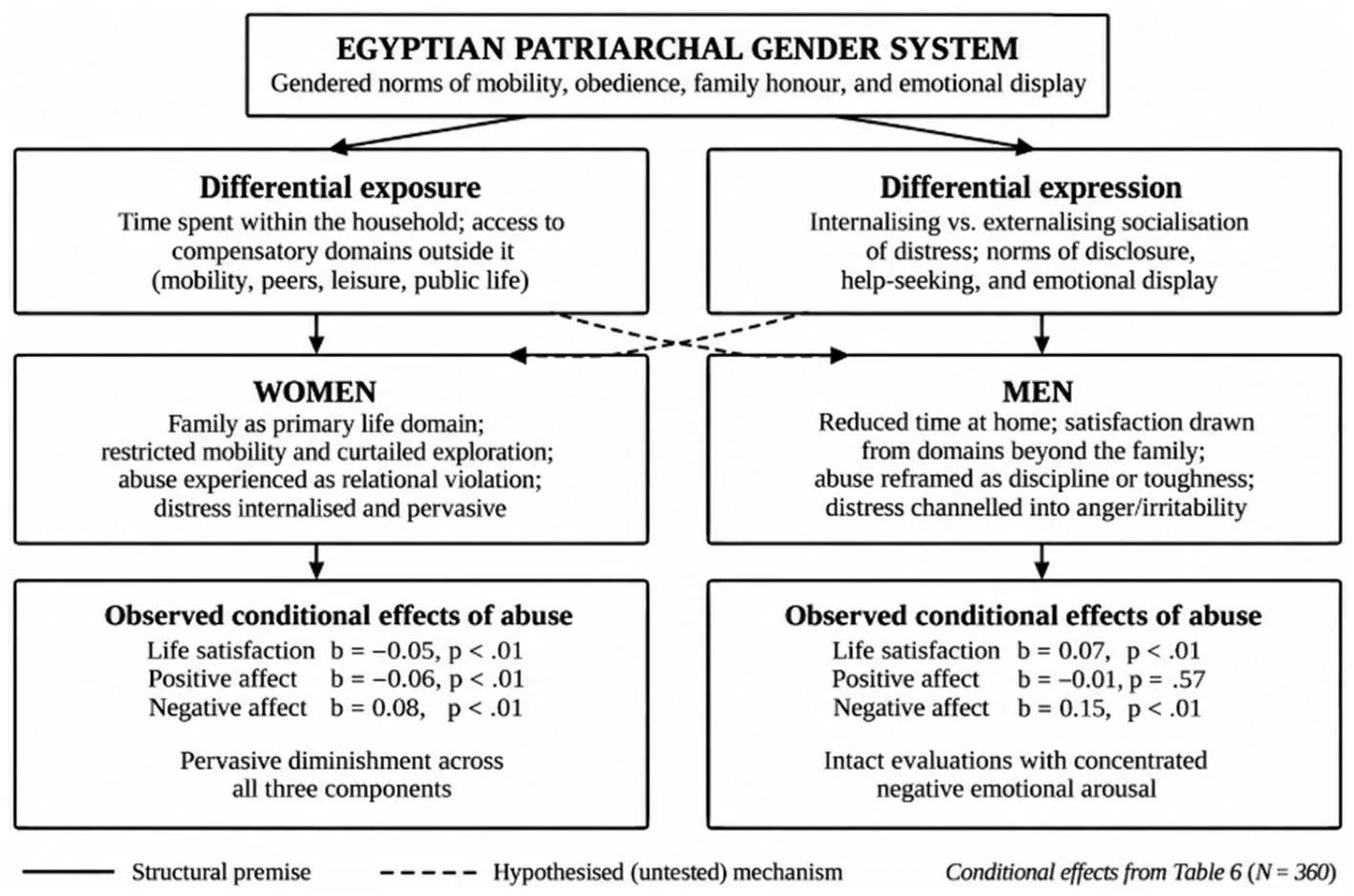
Proposed conceptual model of gendered pathways from parental abuse to subjective wellbeing in the Egyptian context.

H3a was not supported. At the bivariate level, perceived abuse was unrelated to life satisfaction; the moderation analysis revealed why: the association was negative among women but positive among men—a crossover whose opposing slopes cancel in the aggregate. For young Egyptian women, the family is not one life domain among many but the principal arena in which their identities are formed, monitored, and evaluated. Socialization toward relational orientation and familial obedience, restricted mobility under guardianship norms, and the curtailment of the moratorium period for identity exploration leave few alternative domains from which a global evaluation of life can be drawn (Arnett, 2000, 2007; Haj-Yahia, 2000; Shousha, 2020); where disagreement with parents is itself treated as a violation of social and religious norms, and parental control can be disguised as protection of family honor—“indoor aggression” (Ikhtyar for Gender Studies Research Asian-Pacific Resource Research Centre for Ikhtyar for Gender Studies and Research and Asian-Pacific Resource and Research Centre for Women, 2017)—abuse contaminates the very sphere that defines a woman's life, and her life evaluation with it. The positive slope among men is arguably the mirror image of the same gender system rather than an anomaly. First, differential exposure and compensation: young Egyptian men enjoy markedly greater freedom of movement—staying out late, socializing with friends, building daily lives in leisure and public spheres (Shousha, 2020)—which simultaneously reduces time at home, and with it exposure to parental hostility, and supplies compensatory domains of friendship, status, and autonomy that feed life satisfaction independently of the parent–child relationship; the same permissiveness often carries greater material privilege within patriarchal households (Human Rights Watch, 2024). Second, cognitive reframing: masculinity norms invite young men to construe parental harshness as legitimate discipline or preparation for adult responsibility, folding its endurance into an identity narrative that equates toughness with worth (Courtenay, 2000)—a construal that can sustain, or even inflate, global evaluations. Third, differential reporting styles may contribute. H3a was not so much wrong as underspecified: it assumed a single population where the data reveal two, with the same gender system routing the same experience toward opposite evaluative outcomes. Hence, H4a was partially supported: the predicted negative association emerged among women, but the significant positive association among men was not anticipated.

Both H3b and H4b were supported. Higher abuse was associated with diminished positive affect among women only. This is where the exposure dynamic bites hardest. Day-to-day positive emotionality is replenished by autonomous, pleasurable, identity-expanding activity—and it is precisely this that is rationed for young Egyptian women, whose movements are supervised and whose opportunities for exploration are constrained (Arnett, 2000, 2007; Shousha, 2020). A woman living under abusive parental authority therefore has limited access to the experiences that would restore positive mood: the abuse depletes positive affect with little to offset it, consistent with women's documented tendency to internalize family adversity (Maschi et al., 2008) and with evidence that violence exposure undermines flourishing precisely by foreclosing its everyday sources (Lhaksampa et al., 2024). Men's flat slope follows from the same logic run in reverse: their positive affect is substantially generated outside the household, in domains parental abuse does not reach, leaving it effectively decoupled from the parent–child relationship. H4b was supported, but the mechanism is arguably less a matter of women's greater sensitivity than of the gendered geography of where positive emotion is produced.

H3c was supported; H4c was not supported in the predicted direction. Negative affect was the one channel through which abuse registered in both genders—the strongest and most consistent association in the study—indicating that whatever men's evaluative reframing accomplishes, it does not neutralize the emotional charge of being demeaned or struck by one's parents. Contrary to H4c, however, the association was steeper among men, and this reversal is theoretically informative rather than damaging to the framework. First, expression: masculine socialization narrows the acceptable emotional repertoire toward anger and irritability while discouraging disclosure, confiding, and help-seeking (Courtenay, 2000), so men's abuse-related distress is not diffused through sadness, rumination, or social support but concentrates as acute negative emotionality—the externalizing signature documented among maltreated males (Maschi et al., 2008; Topitzes et al., 2011). Second, distribution: women's comparatively flatter slope does not indicate lesser harm but harm dispersed across all three channels—eroded life satisfaction, depleted positive affect, and elevated negative affect—whereas for men the entire impact converges on a single outlet. Read jointly with the life satisfaction crossover, the men's pattern is more consistent with emotional compartmentalization than with genuine resilience: appraisal-level normalization of abuse coexisting with an unprocessed reservoir of negative arousal. This regulatory style is consistent with avoidant strategies in which attachment-related distress is suppressed rather than resolved (Bowlby, 1982), and with evidence that maltreatment heightens rejection sensitivity and relational insecurity into adulthood (Gao et al., 2024)—implying that men's emotional burden, muted in global self-evaluations, may surface most visibly in close relationships. H4c was therefore not supported as predicted: the moderation was significant but reversed in direction. The prediction rested on an assumption of uniform female vulnerability; the data instead reveal channel-specific vulnerability, gendered by where distress is permitted to surface.

Integrating across the three dimensions, the findings cohere with the self-determination framework introduced earlier (Ryan and Deci, 2000): abusive parenting thwarts the basic needs for autonomy and relatedness in both genders, but the gender system determines *which* need is most exposed and *where* the thwarting registers—for women, relatedness within an inescapable family sphere, expressed as pervasive, internalized diminishment of wellbeing; for men, suppressed emotional processing, expressed as concentrated negative affect behind intact evaluations—a pattern summarized visually in Figure 5. This reading is consistent with evidence that maltreatment erodes psychological security (Hedeia, 2018) and predicts enduring mental-health burden from psychological maltreatment specifically (Abo-Al-Ata and Ead, 2019; Xiao et al., 2023). A trauma perspective adds that the chronicity of ongoing abuse—rather than a discrete past event—is precisely the condition under which stress-response dysregulation consolidates (Herman, 1992), lending urgency to its detection during this developmental window. Two implications follow. Clinically, a dual-track approach is indicated: screening Egyptian emerging adult men with life satisfaction or positive affect measures alone would substantially underestimate their burden—negative affect is the sensitive indicator for men—whereas women require assessment across the full wellbeing profile, reinforcing the gender-responsive services outlined in Section 5. Scientifically, because the crossover and the reversed negative-affect moderation were not predicted, these mechanisms—particularly whether men's compensatory domains and cognitive reframing buffer harm or merely mask it, and whether compartmentalization is a stable coping style or a precursor to delayed psychological deterioration—constitute explicit, falsifiable hypotheses for longitudinal replication rather than settled conclusions.

**Figure 5:**
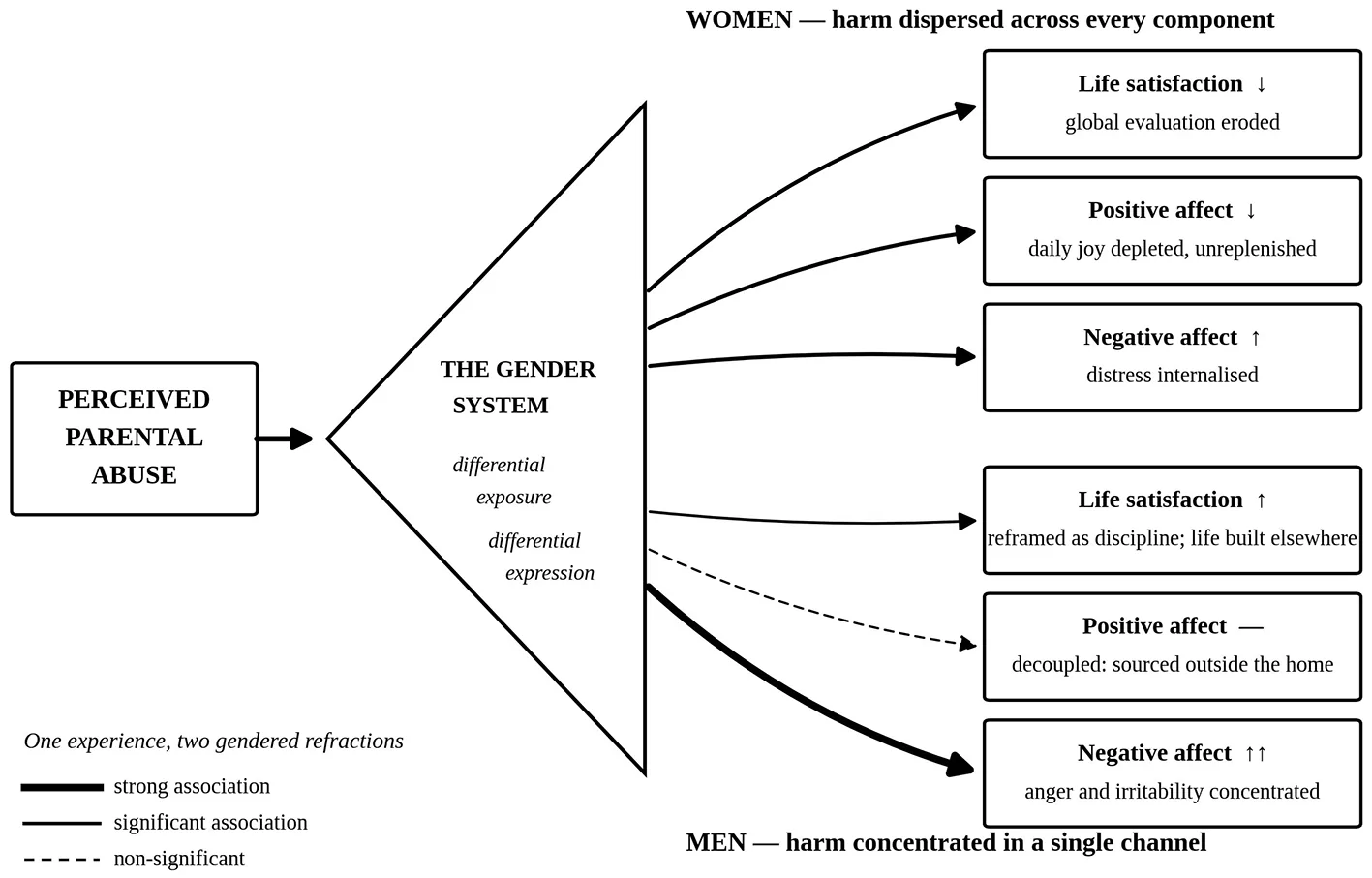
Gendered refraction of parental abuse across the components of subjective wellbeing.

Consequently, these findings not only confirm higher rates of reported abuse among early emerging adult women but also situate that disparity within the same gender system that shapes its consequences. Women's broader vulnerability appears rooted in both emotional susceptibility—reflecting the interplay of biological factors and social conditioning (Moksnes et al., 2010)—and structural position: restricted autonomy and heightened pressure to maintain family honor within hierarchical Egyptian families (Aref, 2020; Human Rights Watch, 2024). Critically, the intersection of gender expectations and family honor also creates an environment in which abuse may be rationalized as necessary discipline or protection, making it harder for victims—women and men alike, though through different rationalizations—to recognize, label, or report such behaviors. This has a direct bearing on the prevalence estimates reported here, which should be read as perceptions filtered through these same gendered interpretive norms.

## 5 Implications

These findings highlight critical areas requiring a multi-pronged and culturally sensitive approach. At the policy and community level, efforts should launch public awareness campaigns in partnership with religious and community leaders to shift the social norms that normalize violent discipline and promote positive parenting, alongside broader community education on child maltreatment and its severe consequences aligned with Egypt's obligations under the United Nations Convention on the Rights of the Child (UNICEF, 2015). Clinically and within institutions, a dual approach is vital. First, routine and sensitive screening for abuse history should be integrated into standard mental health assessments in universities and primary care settings. Second, gender-responsive support services must be established, offering tailored therapeutic programs for populations at higher risk, such as young women, alongside services attentive to men's distinct presentation of distress, given the stronger abuse–negative affect association observed among men. To enable this, professional development for healthcare providers and educators is crucial to enhance their ability to recognize both physical and psychological abuse and provide culturally competent referrals. Concurrently, universities must develop formal protocols to guide academic accommodations and support for students who disclose abuse. For sustained impact, these interventions should be integrated into existing systems. Core components, such as psychoeducation on healthy family dynamics and the long-term effects of abuse, can be embedded into university orientation programs and routine primary healthcare visits for young adults. Finally, at the national level, these efforts require reinforcement through strong child protection legislation and increased funding for youth mental health services targeting emerging adults.

## 6 Limitations of the study and suggestions for future research

Several limitations warrant consideration. First, the reliance on self-reported data may introduce social desirability bias, particularly given the cultural sensitivities surrounding family issues in Egypt. Administration format also varied by session, with some participants completing the survey online and others on paper; format was determined by classroom facilities and logistics rather than by participant characteristics. Differences in responding by format were not assessed, and the possibility that administration mode influenced disclosure cannot be excluded. Relatedly, gender differences in reporting willingness and perception thresholds may have influenced the observed prevalence rates. Men may underreport abuse due to norms of emotional restraint, while women may be more willing to disclose or more sensitive to recognizing abusive behaviors. Additionally, the PPABS measures perceived abuse, which may differ from clinically or independently assessed abuse. Future research should examine concordance between perceived and independently assessed classifications of parental abuse during emerging adulthood. Also, although the PPABS assesses ongoing rather than recalled abuse, it remains a single-administration self-report measure; ecological momentary assessment or diary designs would be needed to capture parental abusive behaviors as they occur.

Second, the use of a university-based convenience sample limits the generalisability of the findings. The results are representative of a university-enrolled, predominantly early emerging adult women subpopulation and may not extend to non-student early emerging adults or to other cultural contexts where different socioeconomic realities, family dynamics, and exposure to parental abuse may apply. Consequently, these estimates should be interpreted as representative of a predominantly young women, university-enrolled sample aged 18–21.

Third, while the developed scale demonstrated a clear two-factor structure, the Covert Abuse factor explained a smaller proportion of the variance compared to the Overt Abuse factor. This may reflect the complexity of measuring covert psychological abuse, which often involves subtle and context-dependent behaviors, a challenge potentially compounded by the smaller number of items dedicated to this dimension. Future research should aim to develop and validate additional items to more fully capture the nuances of emotional and relational abuse. Furthermore, full external validation of the PPABS requires replication with independent samples and examination of convergent validity against established measures of parental abuse.

Fourth, the categorization of continuous abuse scores into high, moderate, and low levels for descriptive prevalence reporting, while empirically grounded in the sample distribution, involves some simplification that may reduce variability. As this categorization was used only for descriptive purposes, findings based on these groupings should be interpreted with caution.

Fifth, given the cross-sectional design, the associations observed between perceived parental abuse and subjective wellbeing cannot be interpreted causally. Longitudinal studies are needed to determine the direction and temporal order of these relationships. Sixth, although age, major specialization, and living arrangement were controlled in the moderation models, other unmeasured factors—such as socioeconomic status and parental education—may contribute to the observed associations. Finally, these limitations highlight critical possibilities for future research. Subsequent studies should strive to recruit more diverse and gender-balanced samples, including non-student populations, to validate prevalence estimates. Moreover, moving beyond the gender differences identified here, an intersectional approach is crucial. Investigating how gender interacts with the unmeasured factors noted above—socioeconomic status and parental education—alongside birth order and family dynamics will lead to a deeper, more contextualized understanding of vulnerability and resilience in the face of parental abuse. In particular, the unexpected positive abuse–life satisfaction association among men and the stronger abuse–negative affect association among men warrant targeted replication.

## 7 Conclusion

This study provides empirical evidence on parent-perpetrated abuse among Egyptian early emerging adults, suggesting significant gender differences in both prevalence and association with subjective wellbeing. The findings demonstrate that abuse during early adulthood is a substantial concern requiring immediate attention, particularly given its disproportionate association with women's life satisfaction and positive affect, alongside the stronger association between abuse and negative affect observed among men. The complex interplay between cultural norms, economic pressures, and gender expectations appears to create unique vulnerabilities that warrant comprehensive, culturally sensitive interventions. The gender-moderated relationship between abuse and wellbeing highlights the need for targeted support services that consider both universal and gender-specific aspects of abuse experiences. Future research and policy initiatives should focus on further investigating these patterns before developing evidence-based interventions that can effectively address these challenges while respecting cultural values and promoting positive developmental outcomes for university-enrolled Egyptian early emerging adults, with future research needed to extend these findings to broader populations.

## Data Availability

The raw data supporting the conclusions of this article will be made available by the authors, without undue reservation.

## References

[B1] Abdeen M. S. Hashim M. A. Ghanem M. M. El-Din N. Y. S. El Nagar Z. M. (2023). Parents' self-reporting of child physical maltreatment (CPM) in a low-middle-income country. BMC Psychiatry 23:506. doi: 10.1186/s12888-023-04947-x 37438727 PMC10339565

[B2] Abdel-Fatah N. A. (2021). Determinants of severe physical disciplinary practices against children in Egypt. Child Abuse Negl. 111:104821. doi: 10.1016/j.chiabu.2020.104821 33257026

[B3] Abo-Al-Ata M. Ead M. N. (2019). Anmā.l-Ata.1016/j.chiabu.2020.104821isciplinary practices against children in Egypt. e country. artifi al-murāhaqah. [Patterns of parental abuse in childhood as risk factors predicting personality disorders in adolescence]. Kafer El-Sheikh University J. Human. Lit. Stud. 1, 118–147. Arabic.

[B4] Abo-Al-Ata M. Ead M. N. (2020). AnmānmāAtaheikh University J in childhood as risk factors predicting personality disord? al-intibar wa furr bar ikh University J i. [Child abuse subtypes during childhood as predicting risk factors of attention deficit/hyperactivity disorder in adulthood: a retrospective-based study]. Kafer El-Sheikh University Arab Studies 19, 436–477. Arabic.

[B5] Ali S. Mohamed K. Kamal M. (2024). “COVID-19 pandemic, child maltreatment, and gender in the MENA region,” in Public Policy in the Arab World: Responding to Uprisings, Pandemic, and War, ed. A. B. Brik (Cheltenham: Edward Elgar Publishing), 92–122. doi: 10.4337/9781035312696.00015

[B6] Al-Katheri Z. M. (2023). Al-isā'ah al-wālidiyya wa 'alāqatuhā bi-al-'unf al-usri li-dā' al-murāhiqin. [Parental abuse and its relation to family violence for adolescents]. J. Adam Univ. Human. Soc. Sci. 4, 415–432. Arabic.

[B7] Al-Mosahar M. A. (2007). Isā'at al-mu'āmalah fi marhalat al-o family violence for adolescents] attention deficit/hyperactivity disorder [Parental abuse in childhood as perceived by female university students and its relation to depression] (master's thesis). King Saud University. Marshallese.

[B8] Aref A. (2020). Parenting Styles and Programs: What Works for Better Parenting in the MENA Region. New York, NY: United Nations Department of Economic and Social Affairs.

[B9] Arnett J. J. (2000). Emerging adulthood: a theory of development from the late teens through the twenties. Am. Psychol. 55, 469–480. doi: 10.1037/0003-066X.55.5.469 10842426

[B10] Arnett J. J. (2007). Emerging adulthood: what is it, and what is it good for? Child Dev. Perspect. 1, 68–73. doi: 10.1111/j.1750-8606.2007.00016.x

[B11] Assaad R. Krafft C. Selwaness I. (2022). The impact of marriage on women's employment in the Middle East and North Africa. Fem. Econ. 28, 247–279. doi: 10.1080/13545701.2021.2007415

[B12] Barsoum G. Ramadan M. Mostafa M. (2014). Labour market transitions of young women and men in Egypt. International Labour Organization. Available online at: https://www.ilo.org/sites/default/files/wcmsp5/groups/public/@dgreports/@dcomm/documents/publication/wcms_247596.pdf (Accessed July 15, 2024).

[B13] Benson P. L. Scales P. C. Syvertsen A. K. (2011). “The contribution of the developmental assets framework to positive youth development theory and practice,” in Advances in Child Development and Behavior, Vol. 41, eds R. M. Lerner, J. V. Lerner, and J. B. Benson (Burlington, MA: Academic Press), 197–230. doi: 10.1016/B978-0-12-386492-5.00008-7 23259193

[B14] Biggart A. Walther A. (2006). “Coping with yo-yo-transitions: young adults' struggle for support, between family and state in comparative perspective,” in A New Youth? Young People, Generations and Family Life, 1st Edn., eds C. Leccardi and E. Ruspini (London: Routledge), 41–62.

[B15] Bowen M. (1978). Family Therapy in Clinical Practice. Northvale, NJ: Jason Aronson.

[B16] Bowlby J. (1982). Attachment and Loss: Vol. 1. Attachment, 2nd Edn. New York, NY: Basic Books.

[B17] Bronfenbrenner U. (1979). The Ecology of Human Development: Experiments by Nature and Design. Cambridge, MA: Harvard University Press. doi: 10.4159/9780674028845

[B18] Cheung G. W. Cooper-Thomas H. D. Lau R. S. Wang L. C. (2024). Reporting reliability, convergent and discriminant validity with structural equation modeling: a review and best-practice recommendations. Asia Pac. J. Manag. 41, 745–783. doi: 10.1007/s10490-023-09871-y

[B19] Clark L. A. Watson D. (1995). Constructing validity: basic issues in objective scale development. Psychol. Assess. 7, 309–319. doi: 10.1037/1040-3590.7.3.309

[B20] Cohen J. (1988). Statistical Power Analysis for the Behavioral Sciences, 2nd Edn. New York, NY: Routledge. doi: 10.4324/9780203771587

[B21] Cohen P. Kasen S. Chen H. Hartmark C. Gordon K. (2003). Variations in patterns of developmental transmissions in the emerging adulthood period. Dev. Psychol. 39, 657–669. doi: 10.1037/0012-1649.39.4.657 12859120

[B22] Constitution of the Arab Republic of Egypt (2014). World intellectual property organization. Available online at: https://www.wipo.int/wipolex/en/legislation/details/15307 (Accessed July 28, 2024).

[B23] Cortina J. M. (1993). What is coefficient alpha? An examination of theory and applications. J. Appl. Psychol. 78, 98–104. doi: 10.1037/0021-9010.78.1.98

[B24] Courtenay W. H. (2000). Constructions of masculinity and their influence on men's well-being: a theory of gender and health. Soc. Sci. Med. 50, 1385–1401. doi: 10.1016/S0277-9536(99)00390-1 10741575

[B25] Crocetti E. Meeus W. (2014). “Family comes first!” Relationships with family and friends in Italian emerging adults. J. Adolesc. 37, 1463–1473. doi: 10.1016/j.adolescence.2014.02.012 24655667

[B26] Deng J. Elmallakh N. Flabbi L. Gatti R. (2024). Labor market transitions in Egypt post-Arab Spring. Middle East Dev. J. 16, 1–25. doi: 10.1080/17938120.2024.2360849

[B27] Dhillon N. Yousef T. Dyer P. (2009). Generation in Waiting: The Unfulfilled Promise of Young People in the Middle East. Washington, DC: Brookings Institution Press.

[B28] Diener E. (1984). Subjective well-being. Psychol. Bull. 95, 542–575. doi: 10.1037/0033-2909.95.3.542 6399758

[B29] Diener E. Emmons R. A. Larsen R. J. Griffin S. (1985). The satisfaction with life scale. J. Pers. Assess. 49, 71–75. doi: 10.1207/s15327752jpa4901_13 16367493

[B30] Duvvury N. Marcos M. O. Gadallah M. Attia S. El-Adly N. Maged W. . (2015). The Egypt economic cost of gender-based violence survey. UNFPA. Available online at: https://egypt.unfpa.org/en/publications/economic-cost-gender-based-violence-survey-egypt-2015 (Accessed August 10, 2024).

[B31] Eagly A. H. (1987). Sex Differences in Social Behavior: A Social-Role Interpretation. Hillsdale, NJ: Lawrence Erlbaum Associates.

[B32] Elghossain T. Bott S. Akik C. Ghattas H. Obermeyer C. M. (2019). Prevalence of key forms of violence against adolescents in the Arab region: a systematic review. J. Adolesc. Health 64, 8–19. doi: 10.1016/j.jadohealth.2018.08.016 30579439

[B33] Elsayed F. K. Muashi L. M. Almoaither M. S. (2022). Prevalence rates of childhood abuse experiences among a sample of Saudi adolescents in light of some demographic variables. J. Educ. Psychol. Sci. 6, 93–113. doi: 10.26389/AJSRP.M040722

[B34] Fishere M. (2021). The ripple effect: a cross-sectional study on the repercussions of child maltreatment among Egyptian college students. Middle East Curr. Psychiatr. 28:11. doi: 10.1186/s43045-021-00147-z

[B35] Fishere M. Habermas T. (2023). “I still remember when…”: narratives of child maltreatment by emerging adults. Journal of Family Trauma, Child Custody and Child Development 20, 57–80. doi: 10.1080/26904586.2022.2036285

[B36] Fornell C. Larcker D. F. (1981). Evaluating structural equation models with unobservable variables and measurement error. J. Mark. Res. 18, 39–50. doi: 10.1177/002224378101800104

[B37] Gao S. Assink M. Bi C. Chan K. L. (2024). Child maltreatment as a risk factor for rejection sensitivity: a three-level meta-analytic review. Trauma Violence Abuse 25, 680–690. doi: 10.1177/15248380231162979 37036152

[B38] Hair J. F. Black W. C. Babin B. J. Anderson R. E. (2019). Multivariate Data Analysis, 8th Edn. Andover, Hampshire: Cengage Learning EMEA.

[B39] Haj-Yahia M. M. (2000). Wife abuse and battering in the sociocultural context of Arab society. Fam. Process 39, 237–255. doi: 10.1111/j.1545-5300.2000.39207.x 10907149

[B40] Hayes A. F. (2022). Introduction to Mediation, Moderation, and Conditional Process Analysis: A Regression-Based Approach, 3rd Edn. New York, NY: Guilford Press.

[B41] Hedeia R. R. (2018). Khuburāt al-isā'ah al-wālidiyya fi al-ultural context of Arab society. urement error. students. so. [Experiences of parental abuse in childhood and its relationship to psychological safety]. Sohag Univ. J. Fac. Arts 2, 197–236. Arabic.

[B42] Henseler J. Ringle C. M. Sarstedt M. (2015). A new criterion for assessing discriminant validity in variance-based structural equation modeling. J. Acad. Mark. Sci. 43, 115–135. doi: 10.1007/s11747-014-0403-8

[B43] Herman J. L. (1992). Trauma and Recovery: The Aftermath of Violence-From Domestic Abuse to Political Terror. New York, NY: Basic Books.

[B44] Hu L. Bentler P. M. (1999). Cutoff criteria for fit indexes in covariance structure analysis: conventional criteria versus new alternatives. Struct. Equ. Model. 6, 1–55. doi: 10.1080/10705519909540118

[B45] Human Rights Watch (2024). Egypt: events 2023. Available online at: https://www.hrw.org/world-report/2024/country-chapters/egypt (Accessed December 15, 2025). doi: 10.46692/9781447318491

[B46] Ikhtyar for Gender Studies and Research and Asian-Pacific Resource and Research Centre for Women (2017). Bodily integrity in the family system in Egypt: perspectives from young people. Available online at: https://arrow.org.my/wp-content/uploads/2017/09/Bodily-Integrity-in-the-Family-System-in-Egypt-Perspectives-from-young-people.pdf (Accessed August 25, 2024).

[B47] Ismail S. M. Atar M. M. (2018). Khuburāt isā'at al-mu'āmalah al-wālidiyya fi al-ily-Integrity-in-the-Family-Systeal-shakhṣiyyah. [Experiences of parental abuse in childhood and its relationship with personality disorders]. J. Fac. Educ., Kafrelsheikh Univ. 18, 313–336. Arabic.

[B48] Jablonski J. F. Martino S. D. (2013). A qualitative exploration of emerging adults‘ and parents' perspectives on communicating adulthood status. Qual. Rep. 18, 1–12. doi: 10.46743/2160-3715/2013.1467

[B49] Jarden A. Roache A. (2023). What is wellbeing? Int. J. Environ. Res. Public Health 20:5006. doi: 10.3390/ijerph20065006 36981914 PMC10049282

[B50] Jyrkiäinen S. Bisset V. (2016). Great expectations, uncertain futures: urban and online encounters with youth, love and marriage in Egypt. AnthropoChildren 6, 1–21. doi: 10.25518/2034-8517.2477

[B51] Kotb A. K. (2012). Khuburāt isā'at al-mu'āmalah al-wālidiyya fi al-riage in Egypt. icating adulthood status. iyyahspectives-from-youn. [Experiences of parental abuse in childhood and its relation to personality disorders among adolescents]. Damanhour University J. Fac. Educ. 4, 50–107. Arabic.

[B52] Kotsiantis S. Kanellopoulos D. (2006). Discretization techniques: a recent survey. GESTS International Transactions on Computer Science and Engineering 32, 47–58.

[B53] Lee M. T. Kubzansky L. D. VanderWeele T. J. (2021). Measuring Wellbeing: Interdisciplinary Perspectives from the Social Sciences and the Humanities. New York, NY: Oxford University Press. doi: 10.1093/oso/9780197512531.001.0001

[B54] Lewis A. Werr P. (2023). Explainer: how deep are Egypt's economic troubles? *Reuters*. Available online at: https://www.reuters.com/world/middle-east/how-deep-are-egypts-economic-troubles-2023-03-03/ (Accessed January 10, 2025).

[B55] Lhaksampa T. C. Grant B. Fix R. L. (2024). The impact of early violence exposure on adolescent flourishing: is there light at the end of the tunnel? J. Prev. 45, 269–285. doi: 10.1007/s10935-024-00765-3 38289562

[B56] Liu C. Rahman M. N. (2022). Relationships between parenting style and sibling conflicts: a meta-analysis. Front. Psychol. 13:936253. doi: 10.37766/inplasy2022.8.0020 36081719 PMC9447430

[B57] Madi A. A. Seifeldin A. Ibrahim A. G. Sultan E. A. (2024). Prevalence of child discipline practices and their associated maternal psychosocial profile in Alexandria. Alexandria J. Med. 45–53. doi: 10.1080/20905068.2024.2321828

[B58] Maly E. G. Grower P. L. Robertson K. E. Haran N. J. Graham-Bermann S. A. (2023). The mental health of emerging adults: hostile home environments and COVID-19. J. Fam. Violence 38, 1577–1589. doi: 10.1007/s10896-022-00478-3 36530538 PMC9735161

[B59] Maschi T. Morgen K. Bradley C. Hatcher S. S. (2008). Exploring gender differences on internalizing and externalizing behavior among maltreated youth: implications for social work action. Child Adolesc. Soc. Work J. 25, 531–547. doi: 10.1007/s10560-008-0139-8

[B60] McDonald R. P. (1999). Test Theory: A Unified Treatment. Mahwah, NJ: Lawrence Erlbaum Associates.

[B61] McKinney C. Brown K. Malkin M. L. (2022). Parental discipline during emerging adulthood: a person-centered approach. Journal of Interpersonal Violence, 37(21–22), NP19424–NP19448. 35343305 10.1177/08862605221084745

[B62] McKinney C. Stearns M. Rogers M. M. (2020). Concordance between parents in offspring maltreatment as perceived by emerging adult college students. Child Abuse Negl. 99, Article 104258. doi: 10.1016/j.chiabu.2019.104258 31739236

[B63] McMahon T. J. (2014). Child maltreatment and emerging adulthood: past, present, and future. Child Maltreatment. 19(3–4) 139–142. doi: 10.1177/1077559514555960 25361790

[B64] Ministry of Health and Population El-Zanaty and Associates, and ICF International. (2015). Egypt Demographic and Health Survey 2014. Cairo: Ministry of Health and Population; Rockville, MD: ICF International.

[B65] Ministry of Planning and Economic Development (2023). Mi 3lopment (l - ‘amal [Egypt in numbers – Work]. Central Agency for Public Mobilization and Statistics. Available online at: https://www.capmas.gov.eg/Pages/Publications.aspx?page_id=5104andYear=23663 (Accessed February 5, 2025).

[B66] Mohammed E. F. (2009). “Values as predictor of subjective well-being among samples of college students,” in Proceedings of the 6th Conference of Psychology: Quality of Life and Psychology (Tanta: Tanta University).

[B67] Moksnes U. K. Moljord I. E. O. Espnes G. A. Byrne D. G. (2010). The association between stress and emotional states in adolescents: the role of gender and self-esteem. Pers. Individ. Dif. 49, 430–435. doi: 10.1016/j.paid.2010.04.012

[B68] National Council for Women (2021). Report on National Strategy to Eliminate Violence Against Women. National Council for Women.

[B69] Potkanski-Palkam M. (2024). Dossier Egypt socio-economic survey 2023. Federal Ministry of the Interior. Available online at: https://www.bmi.gv.at/114/files/Umfrage_Egypt/Dossier_Egypt_Socio_Economic_Survey_2023_nBF_20240408.pdf (Accessed March 20, 2025).

[B70] Rashed D. Allam R. (2022). Gender-Based Violence in Egypt and Morocco: Politics and Policy Making. Barcelona: European Institute of the Mediterranean.

[B71] Reuben A. Moffitt T. E. Caspi A. Belsky D. W. Harrington H. Schroeder F. . (2016). Lest we forget: comparing retrospective and prospective assessments of adverse childhood experiences in the prediction of adult health. J. Child Psychol. Psychiatry 57, 1103–1112. doi: 10.1111/jcpp.12621 27647050 PMC5234278

[B72] Roushdy R. Sieverding M. (2015). Panel Survey of Young People in Egypt (SYPE) 2014: Generating Evidence for Policy, Programs, and Research. Cairo: Population Council. doi: 10.31899/pgy9.1070

[B73] Ryan R. M. Deci E. L. (2000). Self-determination theory and the facilitation of intrinsic motivation, social development, and well-being. Am. Psychol. 55, 68–78. doi: 10.1037/0003-066X.55.1.68 11392867

[B74] Scabini E. Marta E. Lanz M. (2006). The Transition to Adulthood and Family Relations: An Intergenerational Perspective. London: Psychology Press. doi: 10.4324/9780203965221

[B75] Scott K. M. McLaughlin K. A. Smith D. A. R. Ellis P. M. (2012). Childhood maltreatment and DSM-IV adult mental disorders: comparison of prospective and retrospective findings. Br. J. Psychiatry 200, 469–475. doi: 10.1192/bjp.bp.111.103267 22661679 PMC3365274

[B76] Shousha N. (2020). Al-qawālib al-namnambp.111.103267ospebi-al-jindar (al-rujuliyya wa-al-unutha) fi al-mujtama' al-Mi: c. [Gender stereotypes (masculinity and femininity) in Egyptian society]. Psychol. Res. Stud. 16, 226–237. Arabic

[B77] Slep A. M. S. Heyman R. E. Foran H. M. (2015). Child maltreatment in DSM-5 and ICD-11. Fam. Process 54, 17–32. doi: 10.1111/famp.12131 25615555

[B78] Topitzes J. Mersky J. P. Reynolds A. J. (2011). Child maltreatment and offending behavior: gender-specific effects and pathways. Crim. Justice Behav. 38, 492–510. doi: 10.1177/0093854811398578 27667868 PMC5031150

[B79] UNFPA Egypt (2019). Gender-based violence. Available online at: https://egypt.unfpa.org/en/node/22540 (Accessed November 1, 2024).

[B80] UNICEF (2015). Violence against children in Egypt: quantitative survey and qualitative study in Cairo, Alexandria and Assiut. Available online at: https://www.unicef.org/egypt (Accessed September 8, 2024).

[B81] UNICEF (2017a). A familiar face: violence in the lives of children and adolescents. Available online at: https://www.unicef.org/egypt/reports/familiar-face (Accessed September 20, 2024).

[B82] UNICEF (2017b). Challenges: Egypt. Available online at: https://www.unicef.org/egypt/child-protection-0 (Accessed October 5, 2024).

[B83] UNICEF (2018). A profile of violence against children and adolescents in the Middle East and North Africa. Available online at: https://www.unicef.org/mena/media/2826/file/VAC%20in%20MENA.pdf (Accessed October 18, 2024).

[B84] Watson D. Clark L. A. Tellegen A. (1988). Development and validation of brief measures of positive and negative affect: the PANAS scales. J. Pers. Soc. Psychol. 54, 1063–1070. doi: 10.1037/0022-3514.54.6.1063 3397865

[B85] Williams K. (2016). Risk and resilience in emerging adults with childhood parentification (master's thesis). University of Windsor. Scholarship at UWindsor. Available online at: https://uwindsor.scholaris.ca/items/d2275250-0755-439c-b2ce-19400da65dee (Accessed January 15, 2026).

[B86] World Health Organization (2022a). Child maltreatment. Available online at: https://www.who.int/news-room/fact-sheets/detail/child-maltreatment (Accessed November 20, 2024).

[B87] World Health Organization (2022b). Violence against children. Available online at: https://www.who.int/news-room/fact-sheets/detail/violence-against-children (Accessed December 5, 2024).

[B88] World Health Organization (2022c). Mental health: strengthening our response. Available online at: https://www.who.int/news-room/fact-sheets/detail/mental-health-strengthening-our-response (Accessed December 18, 2024).

[B89] Xiao Z. Murat Baldwin M. Wong S. C. Obsuth I. Meinck F. Murray A. L. (2023). The impact of childhood psychological maltreatment on mental health outcomes in adulthood: a systematic review and meta-analysis. Trauma Violence Abuse 24, 3049–3064. doi: 10.1177/15248380221122816 36123796 PMC10594835

[B90] Youssef R. M. Attia M. S. E. D. Kamel M. I. (1998). Children experiencing violence I: parental use of corporal punishment. Child Abuse Negl. 22, 959–973. doi: 10.1016/S0145-2134(98)00077-5 9793719

